# Effects of oligomer toxicity, fibril toxicity and fibril spreading in synucleinopathies

**DOI:** 10.1007/s00018-022-04166-9

**Published:** 2022-03-04

**Authors:** Roberta Cascella, Alessandra Bigi, Nunilo Cremades, Cristina Cecchi

**Affiliations:** 1 Department of Experimental and Clinical Biomedical Sciences, Section of Biochemistry, University of Florence, 50134 Florence, Italy; 2 Institute for Biocomputation and Physics of Complex Systems (BIFI), University of Zaragoza, 50018 Zaragoza, Spain; 3 Department of Biochemistry and Molecular and Cell Biology, University of Zaragoza, 50018 Zaragoza, Spain

**Keywords:** α-Synuclein, Parkinson’s disease, Protein misfolding, Protein aggregation, Amyloid, Toxic oligomers, Lewy bodies, Prion-like, Neurodegeneration, Protein self-assembly

## Abstract

Protein misfolding is a general hallmark of protein deposition diseases, such as Alzheimer’s disease or Parkinson’s disease, in which different types of aggregated species (oligomers, protofibrils and fibrils) are generated by the cells. Despite widespread interest, the relationship between oligomers and fibrils in the aggregation process and spreading remains elusive. A large variety of experimental evidences supported the idea that soluble oligomeric species of different proteins might be more toxic than the larger fibrillar forms. Furthermore, the lack of correlation between the presence of the typical pathological inclusions and disease sustained this debate. However, recent data show that the β-sheet core of the α-Synuclein (αSyn) fibrils is unable to establish persistent interactions with the lipid bilayers, but they can release oligomeric species responsible for an immediate dysfunction of the recipient neurons. Reversibly, such oligomeric species could also contribute to pathogenesis via neuron-to-neuron spreading by their direct cell-to-cell transfer or by generating new fibrils, following their neuronal uptake. In this Review, we discuss the various mechanisms of cellular dysfunction caused by αSyn, including oligomer toxicity, fibril toxicity and fibril spreading.

## Introduction

In protein deposition diseases, such as Alzheimer’s disease (AD) or Parkinson’s disease (PD), different types of aggregated species (oligomers, protofibrils and fibrils) are formed during the process of protein aggregation. Some of these conformers can be toxic to cells/tissues/organs. Numerous experimental studies in cell and animal models of disease [1–4] supported the idea that soluble oligomeric species of different proteins might be more toxic than the larger, fibrillar forms. Furthermore, the lack of correlation between the presence of the pathological hallmark inclusions and disease sustained this issue. Indeed, amyloid-beta plaques and Lewy bodies (LBs) are often found in the brains of individuals without evident signs of AD or PD, respectively. Large fibrillar aggregates are typically harmless for neuronal cells, and the formation of plaques or large aggregated bodies have been suggested to be beneficial once the aggregation process has been triggered [5]. However, we still lack definitive evidence on the nature of the toxic species, mostly due to our failure to detect and define the various protein aggregates.

Synucleinopathies is the general term given to a group of neurodegenerative disorders characterized by fibrillar aggregates of α-Synuclein (αSyn) protein in the cytoplasm of neurons and glia. These disorders include PD, dementia with LBs (DLB), multiple system atrophy (MSA) and pure autonomic failure (PAF) [6]. Glial αSyn filamentous deposits are prominent in MSA and neuronal αSyn inclusions are found in PD, DLB and PAF. Usually, synucleinopathies are clinically characterized by slowness of movement, coordination difficulties or mild cognitive impairment, depending on the distribution of the lesions.

The abnormal processing of the 140 residue protein αSyn plays a key role in the pathogenesis of synucleinopathies [7]. Indeed, the cytoplasmic deposition of amyloid-like aggregates of insoluble αSyn causes the degeneration of key areas of the brain involved in the control of movement or cognition. Under physiological conditions, αSyn functions in its native conformation as a soluble monomer and account for 1% of total central nervous system (CNS) proteins. However, monomeric αSyn is dynamic and can access a large variety of assembly states [8, 9], generating oligomeric and fibrillar species that differ in structure, size and morphology because of its high structural plasticity. Depending on assembly conditions, these states can interconvert over different timescales.

Despite widespread interest, the relationship between αSyn oligomers and fibrils in the aggregation process and spreading remains to be clarified. A wide range of studies clearly suggest that αSyn prefibrillar oligomers, formed at the early stages of the aggregation process, are highly toxic to neuronal cells and play a crucial role in the pathogenesis of synucleinopathies, causing dysfunction in neurotransmitter release, cellular oxidative stress, energy depletion and other pathogenetic pathways ultimately leading to neuronal death [10–12]. Studies in PD-related animal models and post-mortem patient material demonstrate that soluble oligomers and protofibrils of αSyn can accumulate in synapses and axons prior to the onset of disease symptoms. However, some researchers found that αSyn fibrils, which are the most thermodynamically favoured species, are also toxic themselves as contribute to the propagation of the disease [13–17]. Thus, a better understanding of the relative contribution of each stage underlying neuronal dysfunction and neurodegeneration in cellular and animal models may support the development of new therapeutic strategies towards the treatment of synucleinopathies. In this Review, we discuss the various mechanisms of cellular dysfunction caused by αSyn, including oligomer toxicity, fibril toxicity and fibril spreading.

## Oligomer toxicity

### Myriad of structural types of oligomers

The term “oligomer” is widely used to describe aggregated αSyn, although the distinction between oligomeric and fibrillar species needs to be established, with a more appropriate concretion of the properties that need to fulfil an aggregate to be ascribed as either an oligomer or a short fibril. We propose size in combination with the β-sheet content as two of the most critical parameters to distinguish between these two types of aggregated species, with the oligomers displaying a significantly reduced β-sheet content as compared to that of their fibrillar counterparts. It is important to note that αSyn has been proposed to form also α-helical tetramers [18] and higher orders oligomers, mostly upon association with membrane vesicles [19], as functional forms of the protein associated with vesicle fusion in synapsis [20]. These helical oligomeric forms have been, however, reported to be resistant to aggregation [18] and are, thus, considered functional oligomers. Pathological oligomers are associated with the amyloid aggregation process of the protein, and the term “oligomer” in such process encompasses a myriad of species with different sizes, ranging from dimers to high molecular weight species, and with varying degrees of β-sheet contents, from fully disordered structures to oligomers with a significant β-sheet core that starts to resemble that of the fibrils.

There are no current effective methods to detect αSyn oligomeric species in situ, and, consequently, the vast majority of studies that try to elucidate the role of these species in disease have utilised in vitro formed oligomers, which have provided important information on the possible mechanisms and structural features determinant for toxicity in these species, as explained latter in more detail. However, it is crucial that new methods are developed in order to detect specifically these species in vivo and in-patient tissues if we aim to uncover their pathogenic effects. In this context, oligomer conformation-specific antibodies have been used to identify oligomeric species in cells (as examples see [21–23]. Unfortunately, the molecular properties associated with oligomer-specific antibodies are not well understood, and most of them show cross-reactivity between different protein species [24] and among oligomers from different amyloidogenic proteins and peptides [22], as well as other non-amyloidogenic proteins [25]. Alternatively, other groups have combined the use of an antibody against a specific epitope in the sequence of αSyn, which would recognise all forms of the protein, from the monomeric protein to the fibrillar forms, with a strong proteinase K (PK) treatment of the biological sample in what it has been referred to as PK paraffin-embedded tissue blot (PK-PET blot) [26]. The PK treatment guarantees that only PK-resistant protein forms are detected by the antibody, being the mature fibrillar αSyn, thus, the most likely species detected by this method. A different strategy, this time based on the proximity ligation assay (PLA) methodology, was developed by the Alegre-Abarrategui group [27], in which the same antibody that recognises αSyn (syn211) is conjugated to a short oligonucleotide. When two conjugated antibodies are in proximity, when bound to an αSyn oligomer or fibril, the oligos become ligated, acting as a primer for DNA polymerase to generate concatemeric sequences through a rolling-circle amplification process using complementary fluorescently labelled oligos. This method allows up to 1000-fold amplified signal that is tethered to the protein complex. Using this approach, the authors described the presence of abundant oligomers diffusely located around the LBs in the medulla and cingulate cortex, which colocalised with pathology in PD post-mortem brain tissue. However, again, this method is not generally able to discriminate between oligomers and fibrils, presenting only a two-fold higher signal for the oligomers analysed, with respect to fibrils, according to the authors [27]. Other methods to ascribe biological effects to oligomeric αSyn species have used cells expressing non-natural αSyn variants bearing specific single-point mutations designed to accumulate oligomeric species distinct to the fibrillar forms [4] or αSyn bimolecular fluorescence constructs that form oligomers [28]. Overall, these studies show that overexpression of oligomer-prone αSyn variants induces neurodegeneration in the substantia nigra pars compacta of rats [4, 29], while fibrillation-prone variants did not display toxicity [4]. While these studies are essential to understand the nature of the toxic species and their roles in disease, they are still unable to provide detailed structural information of these species, which hampers the establishment of structure–toxicity relationships and, therefore, the rational design of molecules capable of preventing or reverting their pathological effects.

In vitro, two main structural subgroups of oligomers have been reported to be formed during fibril formation: a subgroup of primarily disordered oligomers that are initially formed and a subgroup of more compact, stable and proteinase K resistant oligomers with partial β-sheet structure [12, 30]. By the use of single-molecule experiments, we demonstrated that the main mechanism of fibril formation by αSyn under the most commonly used in vitro conditions follow a nucleation-conversion-polymerization kinetic model [12]. In this model, monomers initially establish the first contacts, typically when the monomeric protein is adsorbed into a hydrophobic/hydrophilic interface (heterogeneous nucleation), forming the early, primarily disordered oligomers, which then suffer a slow structural conversion into oligomers containing β-sheet structure, which further elongate, increasing their β-sheet content, until they rich the fully formed fibrillar structure [30]. The increase in β-sheet structure in these prefibrillar oligomers is likely to be concomitant with an increase in their ability to recruit monomeric protein and grow in size (elongation). The small fibrillar species then generated represent the active nuclei for elongation and multiplication through secondary processes, such as secondary nucleation and fragmentation, speeding up fibril formation exponentially [31, 32].

Interestingly, Gosh et al. [33] have proposed that αSyn forms a helix-rich oligomeric intermediate during fibril formation, which can populate ca. 50% of the protein species at the middle point of reaction and, when isolated, can seed the formation of fibrils. In contrast, under similar in vitro conditions (in the absence of lipid vesicles or surfactant molecules), other studies only report the population of very low concentrations of oligomers, in some cases distinguishing distinct structural subgroups with either a high degree of disorder or with some partial β-sheet structure [12, 24, 34]. It is possible that a subset of oligomers formed during the transition of the protein from the unfolded monomeric to the β-sheet fibrillar forms contains some α-helical structure, although it is surprising to believe that the population of such subset could reach such high protein mass percentages at any time of the reaction at the typical conditions used to induce αSyn fibril formation [35]. Further studies would be required to clarify these initial observations.

The typical low populations of oligomers accumulated during the reaction of fibril formation and their heterogeneous and transient nature, has generally prevented the detection of such species in both cellular (as we do not have also specific good probes for their detection) and in vitro experiments. As a consequence, there are not yet detailed structural studies at atomic resolution for an αSyn oligomeric form (again oligomer referring as a different structural entity with respect to fibrils) in comparison to a large number of structures of different polymorphs of fibrils already available [36]. Kinetically-trapped αSyn oligomeric species isolated either by the use of compounds able to inhibit their conversion to fibrils or by the use of alternative amyloid pathways with particularly high energetic barriers between oligomeric and fibrillar forms of the protein are particularly useful for obtaining insightful information about these otherwise transient species [30, 37].

Polyphenol(−)-epigallocatechingallate (EGCG), baicalein, nicotine, dopamine, H_2_O_2_ and 3,4-dihydroxyphenylacetic acid have been frequently used to promote αSyn oligomerization in vitro [38–43]. These treatments generally result in the oxidative modification of αSyn which leads to the accumulation of oligomers, typically by modifying the structure of the oligomers and preventing them to continue their evolution to mature fibrils. These modified oligomers are usually disordered and non-toxic (see [30] for a more detailed review of different structural types of αSyn oligomers). A different structure, however, has been proposed for the oligomers formed in the presence of the reactive aldehyde 4-oxo-2-nonenal (ONE) or 4-hydroxy-2-nonenal (HNE), which were found to covalently modify αSyn in vitro and induce the formation of stable β-sheet-rich oligomers with neurotoxic properties [44, 45].

An alternative strategy used to generate stable αSyn oligomeric samples involves the induction of particular mechanisms of amyloid self-assembly. We have recently observed that αSyn can form amyloid aggregates with similar β-sheet content to that of the typical fibrils, with all the structural hallmarks of amyloid structures, but with smaller sizes and with a preference for an antiparallel β-sheet arrangement, in contrast to the parallel β-sheet architecture observed for the fibrils generated under the typical in vitro conditions used to trigger αSyn self-assembly. We established that this alternative amyloid β-sheet structure was preferred when the process of self-assembly was triggered by homogeneous nucleation and that this type of nucleation was favoured under limited hydration conditions. Indeed, we noticed that the formation of amyloid aggregates rich in intermolecular antiparallel β-sheets under limited hydration conditions was already reported for other amyloidogenic peptides, and a multitude of a priori non-amyloidogenic proteins belonging to different structural classes, as well as disordered peptides such as poly(l-lysine) [46–50]. Under highly hydrating conditions, primary nucleation in αSyn is slow, being facilitated by the presence of hydrophobic/hydrophilic interfaces such as the air/water interface (heterogeneous nucleation). The pre-nucleus of the amyloid structure formed at these nucleation-active surfaces would inevitably adopt a parallel intermolecular β-sheet arrangement given the restrictions in the disposition and orientation of the polypeptide chains anchored through their N-terminal amphipathic region to the interface. Under conditions of poor water activity, similar to those found in the interior of protein droplets generated by liquid–liquid phase separation, however, the desolvation energy barrier is significantly reduced, and nucleation can occur very rapidly in the bulk of the solution (homogeneous nucleation), giving rise to structurally distinct amyloid polymorphs, as there is no restriction in the orientation of the protein molecules in the bulk [51].

The antiparallel intermolecular β-sheet structure has been also previously observed in stable, particularly toxic oligomers of αSyn and other amyloidogenic systems and has been proposed to be distinctive of these toxic species [52–55]. These oligomers have been suggested to be off-pathway but, in the light of our new findings, they are best described as amyloid prefibrillar oligomers generated by homogeneous nucleation under limited hydration conditions. We noticed that a significant number of reported protocols to generate stable β-sheet oligomers include a lyophilization step or the peptide/protein stock is lyophilized, which suggests that similar types of oligomers to those generated under limited hydration conditions (with antiparallel β-sheet core) are formed.

These stable oligomers have been reported to represent good models to investigate the structural and biological properties of prefibrillar amyloid oligomers as they are homologous to the transiently populated parallel β-sheet oligomers that are formed during fibril formation through heterogeneous nucleation at the typical conditions used for in vitro fibril formation (air/water interface under shaking conditions) [54, 56]. However, not all the properties are similar or homologous, as the kinetically trapped nature of these species implies that they are deficient in elongating [57], in contrast to the transient parallel β-sheet oligomers, which are consequently difficult to trap. Importantly, this type of oligomers seems biologically analogous to the oligomeric species formed in cells, being the cellular effects that they induced (except seeding and propagating) similar to those observed in a PD cellular model obtained from iPSC-derived neurons from a patient with a triplication in the SNCA gene (the gene that encodes for αSyn) [58, 59].

These kinetically trapped, partially formed β-sheet oligomers present half of the β-sheet content (~ 30%) to that typically observed in the matured fibrils (~ 60–65%, either in the parallel or antiparallel β-sheet fibrillar structures) and in average contain ca. 30 mers (although distribution of 15–40 mers is generally observed) [54]. The structural homogeneity of the oligomeric samples obtained following well-established protocols [52, 54, 60] and the remarkably slow elongation and disaggregation rates of this type of oligomers [54, 61] have allowed their structural characterization in more detail by a wide variety of techniques: fluorescence [62] and infrared spectroscopy [52], mass spectrometry [63], SAXS [34, 60], NMR [64] or cryo-EM [54]. A consensus among all the structural studies can be extracted. These oligomers show a cylindrical/ellipsoidal morphology, similar to that previously reported for toxic prefibrillar oligomers [34]), with a diameter of ca. 10 nm, that coincide with the diameter found for αSyn mature fibrils. However, in contrast to the αSyn fibrils whose structure has been determined up to date, these oligomers show a hollow core, suggesting that the interactions between β-sheets are predominantly hydrophilic and mediated by water molecules. This structural arrangement results in a significant fraction of hydrophobic residues exposed to the solvent at the surface of the oligomer structure [54], which combined with a flexible, partially formed core structure, explains the particular ability of these species to interact with the interior of the lipid bilayers and disrupt cellular membranes [64]. In contrast, the fibrillar forms of the protein have a highly rigid β-sheet core [65] which impedes its insertion into the lipid membranes [11]. The exposure of hydrophobic residues and hydrogen-bond unsatisfied amino acid groups on the surface of these oligomeric species likely results also in the aberrant and promiscuous interaction of these species with other cellular components, which could disrupt their correct biological functions.

### Mechanisms of toxicity

Accumulating evidence indicates that αSyn oligomers are toxic. They have been reported to locate at the degenerating regions in PD in vivo models [66, 67] and different reports have shown direct experimental evidences for toxic properties of oligomers in cells [28], with impaired αSyn variants for fibril, but not oligomer formation, showing the greatest toxicity [3, 4]. In addition, certain in vitro generated oligomers were found to be particularly neurotoxic in vivo in different model organisms [3].

Several observations suggest that αSyn oligomers, similarly to other prefibrillar oligomers from other amyloidogenic proteins, can disrupt cell membranes, being this effect a primary event in αSyn aggregation toxicity [59, 64, 68–70]. The most likely mechanism is the direct interaction of such species with the cell membrane and the insertion of part of their amyloid core into the lipid bilayer [64], with the concomitant membrane disruption and aberrant ion efflux/influx in the cell [11, 68]. Such ion dyshomeostasis can promote oxidative stress by increasing reactive oxygen species (ROS) production and decreasing endogenous glutathione [59], but also abnormal calcium influx [58], which has been related to mitochondrial dysfunction [71].

Interestingly, in some of these studies, identical effects were observed in both inducible pluripotent stem cells (iPSC)-derived neurons bearing SNCA triplication that generates aggregates that promote oxidative stress and mitochondria dysfunction, as well as in primary rat co-cultures of neurons and astrocytes exposed to in vitro generated kinetically trapped αSyn oligomers with β-sheet structure of the type characterised in [54, 58, 59, 72]. More recently, a direct interaction between αSyn oligomers and ATP synthase has been reported, which seems to result in the impairment of complex I-dependent respiration, the opening of the permeability transition pore and mitochondrial swelling, eventually causing cell death [72]. In agreement with these findings, earlier studies already showed αSyn aggregates accumulation in mitochondria in neurons in culture and in vivo, impairing complex-I function [71, 73]. Overall, different studies on the toxic effect of αSyn oligomers suggest that there are multiple mechanisms by which these species can induce mitochondrial dysfunction both in neurons and astrocytes [74, 75].

αSyn oligomers are formed inside cells but can also be internalised if present in the extracellular media by processes mediated in part by endocytosis [57, 76] and in part by other mechanisms [11]. The interaction of some types of oligomers with the plasma membrane of cells causes their perturbation, as pointed before, initiating a cascade of toxic effects [11]. In the interior of neurons, these oligomers are accumulated which in turn causes endoplasmic reticulum (ER) stress [77, 78] and proteostasis system dysfunction, apparently impairing both the proteasome and the autophagy-lysosomal degradation pathways [79, 80]. αSyn oligomers have been reported to directly interact with some units of the proteasomal system, blocking the entry of other substrates and increasing the accumulation of misfolded proteins. At the same time, cellular insults such as oxidative stress have been shown to increase αSyn oligomer formation [81], further exacerbating an aberrant pathological feedback, which could be particularly deleterious in the high energy-demanding dopaminergic neurons, which also present high basal levels of oxidative stress [82].

**Fig. 1:**
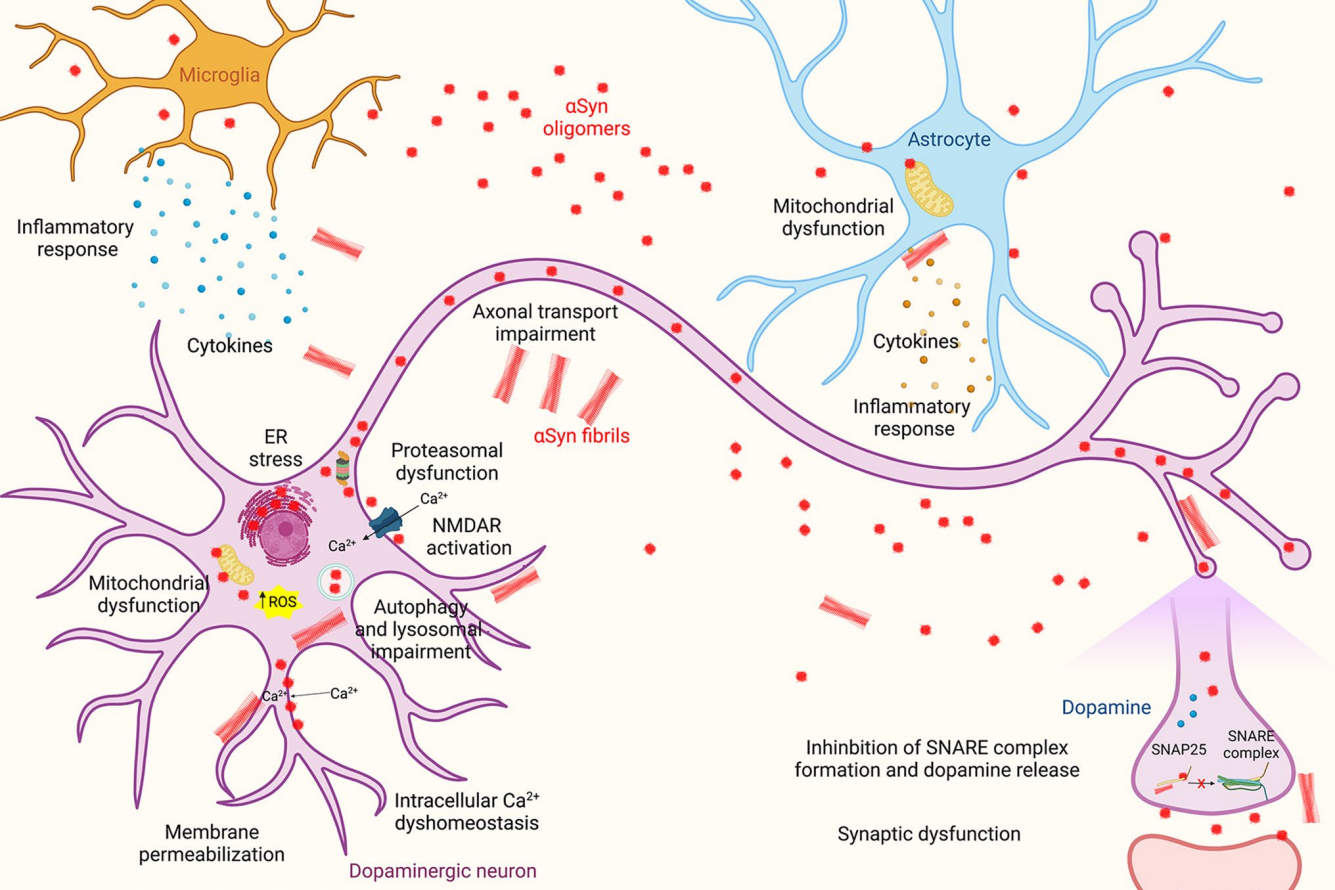
Primary mechanisms of αSyn aggregate toxicity. A myriad of mechanisms has been proposed for the induction of toxicity by αSyn oligomers and fibrils. In this schematic representation have been highlighted some of the most well-documented mechanisms, as explained in the text. Created with BioRender.com

In addition, other toxic effects have been associated with αSyn oligomers, including activation of pro-inflammatory cytokines in astrocytes and microglia [83]. Extracellular aggregated αSyn has been reported to interact with toll-like receptors on glial cells and trigger inflammatory responses which eventually cause neuronal death [83, 84]. In addition, αSyn aggregated species have also been reported to impair axonal transport in neurons [85] and promote synaptic dysfunction [86, 87] through aberrant interactions with NMDA receptors [88, 89]. Indeed, some studies have shown an increased negative impact on neuronal signalling for some types of oligomers with respect to either monomeric or fibrillar forms of αSyn [2]. The different neurotoxic pathways and cellular dysfunctions evoked by αSyn oligomers are reported in Fig. 1.

The multiplicity of the type of molecules that αSyn oligomers have been reported to directly interact with or indirectly affect could be associated in part with the variability of types of oligomers used in the different studies and the terminology used in some of these studies, where the name oligomers is used indiscriminately to also include fibrillar aggregates. Most studies on oligomers have not characterised the species of oligomers in detail, so it is extremely difficult to extract firm conclusions about the general role of these species in pathology. In any case, the amount of different studies on the toxic effects of αSyn oligomers reflects the diversity of aberrant interactions of these species to multiple cellular components, with detrimental consequences for the cell. The ability of these species to interact aberrantly with such multiple types of molecules is likely a consequence of the particular structural attributes of these species, as explained before. Such attributes are likely to be common to other hydrophobic, partially β-sheet formed oligomers from other amyloidogenic proteins [22, 90, 91], although structural peculiarities among the different types of oligomers probably imply specific interactions and mechanisms of toxicity for distinct types of oligomers. It is very important, therefore, to classify structural groups of oligomers and identify their properties and effects to clarify their role in disease.

Synucleinopathies, as well as other amyloid-associated neurodegenerative diseases, have a distinct progression pattern for neurodegeneration in the brain concomitant with the appearance of αSyn pathology, suggesting a neuropathological propagating role of αSyn.

αSyn oligomers can induce neurotoxicity in a variety of ways and can be propagated from cell to cell, properties that have made the scientific community to consider them as key players in the aetiology of synucleionopathies. Given that a primary event of oligomer toxicity involves perturbation of neuronal membranes, a number of molecules and strategies have been designed to target the interaction of oligomers with these cellular structures, which, indeed, have been reported to elicit beneficial effects in different cellular and animal models [56, 92–97]. Some of these molecules are being tested as potential therapeutic agents.

Immunotherapy could be an alternative method to target αSyn pathology. In this respect, monoclonal antibodies developed to target oligomeric αSyn were used to treat transgenic mice expressing high levels of αSyn and the researchers found reduced levels of αSyn in the central nervous system (CNS) [98]. Following a similar idea, Spencer et al. [99] used lentivirus-mediated delivery of oligomer-selective nanobodies (single chain antibodies) to increase the intracellular presence of antibodies inside the CNS and shown to reduce αSyn pathology and motor symptoms in transgenic mice. These findings together with similar results obtained from other groups have prompted clinical studies of αSyn immunotherapy in humans. Although the first results recently obtained from these clinical studies are less encouraging than expected, efforts in improving the delivery of antibodies inside neurons in the CNS, as well as in developing antibodies that truly target specifically the toxic aggregated forms of αSyn that form in vivo, without affecting the monomeric, functional form of the protein, should continue to really validate this approach as an effective therapy.

At the same time, increased levels of αSyn oligomers in brains and CSF have been measured in individuals with LB-related pathologies compared to control subjects [26, 100–105], suggesting that αSyn oligomers could be relevant biomarkers present in human biofluids which could guide earlier diagnosis and more effective prognosis, as well as for therapy evaluation studies in synucleionopathies. To detect these species in patient samples, efficient and highly specific detection methods are required. Some current approaches to detect aggregated αSyn in human biofluids use a PMCA (amplification cycle) approach. Interestingly, this technique has been recently shown to be able to discriminate patients with PD from patients with MSA [106]. The approach consists of multiplying primarily the αSyn aggregates with the highest elongation capabilities and, therefore, will enrich the sample in aggregates with the faster rates of elongation, which are likely to consist of particular types of fibril polymorphs. It is yet unclear whether these approaches are effective in detecting oligomeric species, being those different species with respect to fibrils. In any case, this approach shows a great potential for differential diagnosis and it represents a huge step towards the specific and earlier diagnosis to distinct types of synucleinopathies. Other methods to identify and quantify αSyn oligomers as biomarkers in CSF or blood are based on ELISA detection, using primarily the monoclonal αSyn antibody 211 (epitope in C-terminal region of αSyn) for capture and detection, so they cannot discriminate between oligomeric and fibrillar forms or either different structural types of oligomers. In addition, these approaches can potentially cross-react with monomers if they are associated with vesicles, or other protein complexes. In this context, molecules that can bind specifically the toxic species of αSyn, even under conditions of a large excess of monomeric protein [56], can be very useful for developing diagnostic tools.

## Fibril toxicity

### αSyn fibrils

The accumulation of amyloid-like αSyn fibrils as intracellular inclusions in neurons (LBs) is one of the hallmarks of PD [107–111]. However, whether αSyn fibrils and LBs are the cause or consequence of synucleinopathies remains the subject of scientific debate. Indeed, the molecular and cellular mechanisms triggering αSyn misfolding, fibrillization, and LBs formation appeared unclear and are only partially characterized.

Monomeric αSyn self-assembly finally leads to the formation of highly compact and ordered fibrils that show the common amyloid features, such as the ability to bind Thioflavin T, the presence of a β-sheet structure and an elongated filamentous morphology [45, 52] and that have been found to be morphologically and tinctorially almost identical to those extracted from patients [112]. Several techniques have been employed for the structural characterization of αSyn fibrils, such as electron microscopy (EM), hydrogen exchange mass spectrometry, solid-state nuclear magnetic resonance (ssNMR), or electron paramagnetic resonance (EPR) [30]. Many accumulated evidences indicate that the core structure of αSyn fibrils generally includes residues 30–110, is resistant to proteases and completely buried [113–116], whereas both the C-terminus (residues 100–140) and the N-terminal region of the protein (residues 1–30) remain largely unstructured, although both regions seem to be involved in the interactions between protofilaments [114, 117]. Furthermore, αSyn fibrils adopt an antiparallel in-register β-sandwich fold.

All αSyn species are in equilibrium in solution and their concentration and life span depend on the environment of those molecules. Thus, αSyn conformers form and grow at different rates under different environmental conditions, leading to a variety of fibrillar polymorphs [118], which vary in the number and disposition of the protofilaments in the fibrils and in the strength of the amino acid interactions responsible for the β-sandwich fold [114, 116, 119]. EM studies have shown that, independently of the fibril polymorphism, several αSyn fibrils possess a low electron density region in the center of their structure [114], as they are formed by many protofibrils that, interacting with each other, form a tubular-like ultrastructure, in both twisted and straight fibrils [30]. Cryo-EM studies revealed that the residues 50–57 of αSyn fibrils are located at the interface of the interacting protofilaments. The various αSyn fibril polymorphs have been observed both in vitro and in vivo. In particular, negative-stain EM images of αSyn fibrils extracted from the brain of PD and MSA patients revealed the presence of 10-nm-wide straight or twisted filaments and a minor population of 5-nm-wide straight filaments [111]. Additional EM studies of recombinant αSyn fibrils confirmed the presence of similar fibrillar polymorphs [114].

To clarify the molecular mechanisms by which αSyn generates distinct fibrillar aggregates and triggers neurotoxicity, several structural analyses of αSyn fibril polymorphs have been carried out. The NAC domain of αSyn is the hydrophobic core of the protein formed of 12 amino acids that are responsible for its fibrillogenesis [120]. More than six different amyloid fibril structures of αSyn have been solved by cryoEM or ssNMR [36, 121], and all the fibril cores contain the NAC region [120]. Notably, between 50 and 70% of the 140 residues of αSyn are not involved in the cross-β amyloid core. In particular, structures of the preNAC and NAC regions have been resolved by micro-electron diffraction (microED) [122], revealing the presence of a pair of tightly mated in-register β-sheets forming a steric zipper. Moreover, ssNMR analysis of recombinant αSyn revealed a Greek-key β-sheet motif in the hydrophobic core of a single fibril filament [119], where salt bridges (E46-K80), a glutamine ladder (Q79), and hydrophobic packing of aromatic residues (F94) contribute to the stability of the in-register β-sheet. The different αSyn fibril polymorphs were found to exhibit different toxicity and seeding properties both in vitro [123] and in vivo [124]. Recent studies demonstrated that brain-derived αSyn fibrils from different synucleinopathies are distinct in seeding potencies [125]. However, additional studies are needed to elucidate the differences between αSyn fibril polymorphs, and to develop new therapeutic approaches targeting αSyn aggregation and seeding.

Recent NMR and circular dichroism (CD) data have shown that the β-sheet core of the αSyn fibrils are unable to establish persistent interactions with either the surface or the internal regions of the lipid bilayers, with the interactions restricted to the binding of the disordered N-terminal region of αSyn onto the surfaces of the lipid bilayers [11]. Accordingly, at early incubation time, αSyn fibrils appear to be largely localized at the surface of the plasma membrane. Furthermore, the total quantity of fibrils bound to the cell membrane was not found to correlate with the degree of cell dysfunction or αSyn penetration [11]. Therefore, the association between fibrils and cellular membranes is not sufficient to account for the toxicity of these species.

Stress conditions cause the imbalance of neuronal proteostasis leading to pathological situations [126, 127]. Even if the aggregation of αSyn is independent of cellular proteostasis, the life span and concentration of the aberrant species depend on cellular proteostasis. Furthermore, it is well known that point mutations (A30P, E46K, H50Q, G51D, or A53T) and gene duplication/triplication within SNCA, the gene encoding αSyn, are associated with increased aggregation propensity and familial early-onset forms of PD [128–132]. Indeed, these gene alterations increase the number of possible αSyn conformations and life span, thus favouring its aggregation. In particular, A30P and A53T mutations promote fibril formation more rapidly than the wild-type αSyn protein and are more prone to αSyn toxicity. Although LBs are characterized by the presence of αSyn fibrils with β-sheet conformation [122], in vitro studies have shown that A30P and A53T αSyn mutations induce the formation of oligomers with respect to fibrils [133]. In addition, both wild type and mutants A30P and A53T αSyn can form insoluble β-sheet structured fibrillar aggregates at physiological temperature.

Recent EM and proteomic studies have shown that LBs are formed by a complex mixture of aggregated forms of αSyn and numerous other proteins, lipids, parts of membranes and organelles [134]. However, the role of these proteins in αSyn aggregation and the biogenesis of LBs remains poorly understood. Recent results demonstrated that the formation of αSyn fibrils occurs before the formation of LB-like inclusions [135], but what triggers αSyn fibrillization remains elusive. Previous studies have focused on the role of several proteins as modifiers of αSyn aggregation, inclusion formation, and toxicity. Recently, the aminoacyl tRNA synthase complex-interacting multifunctional protein 2 (AIMP2) has been pointed as an essential promoter of αSyn aggregation, inclusion formation and cytotoxicity [136]. Although AIMP2 has been suggested to be toxic to dopamine neurons, it showed a self-aggregating property acting as a seed to increase αSyn aggregation via specific and direct binding to the αSyn monomer. AIMP2 aggregates have been found to bind to monomeric αSyn, colocalizing with pS129 αSyn aggregates and inclusions in several models of synucleinopathies. These findings, however, lack of supporting data on the role of AIMP2 in aSyn aggregation or inclusion formation, suggesting that other mechanisms need to be discovered.

### Mechanisms of toxicity

The αSyn encoding gene, SNCA, is a major genetic risk locus for idiopathic and familial PD [137–139]. αSyn is found both in the cytosol and in the extracellular interstitial fluid. The oligomeric species were observed in the cerebrospinal fluid (CSF) of PD patients [140], promoting the seeding and aggregation of monomeric αSyn [141, 142]. The study of neuronal degeneration induced by intracellular αSyn is limited by the difficulties of generating in vitro intracellular aggregated species. On the other hand, the toxicity of extracellular species can be more easily monitored. Indeed, αSyn pathology can be triggered in experimental animals after intracerebral, intramuscular, or intravenous inoculation of brain homogenates from affected mice and patients, or recombinant fibrillar αSyn [15, 125, 143–146]. After neuronal transplantation in the brain of PD patients, grafted neurons showed LB-like inclusions [147, 148]. Furthermore, the injection of aggregated αSyn was found to cause the loss of nigral dopaminergic neurons [15, 144, 149].

The most widely accepted paradigm postulates that prefibrillar oligomers, with respect to mature fibrils, represent the neurotoxic agents in PD [4, 133]. Nevertheless, some researchers found that αSyn fibrils are toxic themselves, because of their ability to bind and permeabilize the plasma membrane, thus inducing several alterations in cultured cells, such as Ca^2+^ dyshomeostasis [150] and mitochondrial dysfunction [151]. However, a direct quantitative in vivo comparison of the neurotoxic potential of different αSyn conformers is lacking. Fibrillar forms of αSyn have been reported to be both toxic and non-toxic [12, 40, 45, 68]. However, αSyn fibrils can contribute to neurodegeneration by different pathways: alteration of ionic homeostasis, seeds of soluble αSyn into higher molecular weight aggregates [23], perturbation of cellular proteostasis [126, 127], alteration of the integrity or function of cytosolic organelles [152], and activation of chronic inflammation [153–155]. Furthermore, there is evidence that the formation of fibrils, rather than toxic species themselves, mediates αSyn toxicity [109]. In particular, the fibrillary species, as opposed to the oligomeric forms, have been shown to propagate the pathology by recruiting endogenous αSyn when injected into animal models [16]. Indeed, αSyn fibrillar species generated in vitro cause neuronal degeneration and trigger the aggregation of endogenous αSyn several months after injection into the central nervous system (CNS), similar to brain homogenates from PD or MSA cases [14, 16, 17, 124].

Preformed αSyn fibrils were also found to cause the loss of the proteins VAMP2 and SNAP-25 that are associated with the soluble NSF-attachment protein receptor (SNARE) complex in primary neuronal cultures. These fibrils also reduced the levels of two proteins involved in the synaptic vesicles (CSPa and synapsin-2) [17]. The researchers further investigated the impact of the accumulation of these pre-formed αSyn fibrils in neuronal degeneration. In particular, they found that impaired hippocampal network activity occurred much earlier than the loss of synaptic proteins, suggesting that αSyn pathology can have a major impact on the coordination of neuronal communication and connectivity [17]. αSyn preformed fibrils also induced robust inflammatory transcriptional signalling in human midbrain astrocytes [156]. The major neurotoxic effects of αSyn fibrils are represented in Fig. 1.

The deleterious effects of αSyn may exist independently of endogenous αSyn. Indeed, neurotoxicity can be differentially affected by the type of αSyn aggregates added to cells and oligomer-induced cell death did not promote the aggregation of endogenous αSyn [11, 68].

**Fig. 2:**
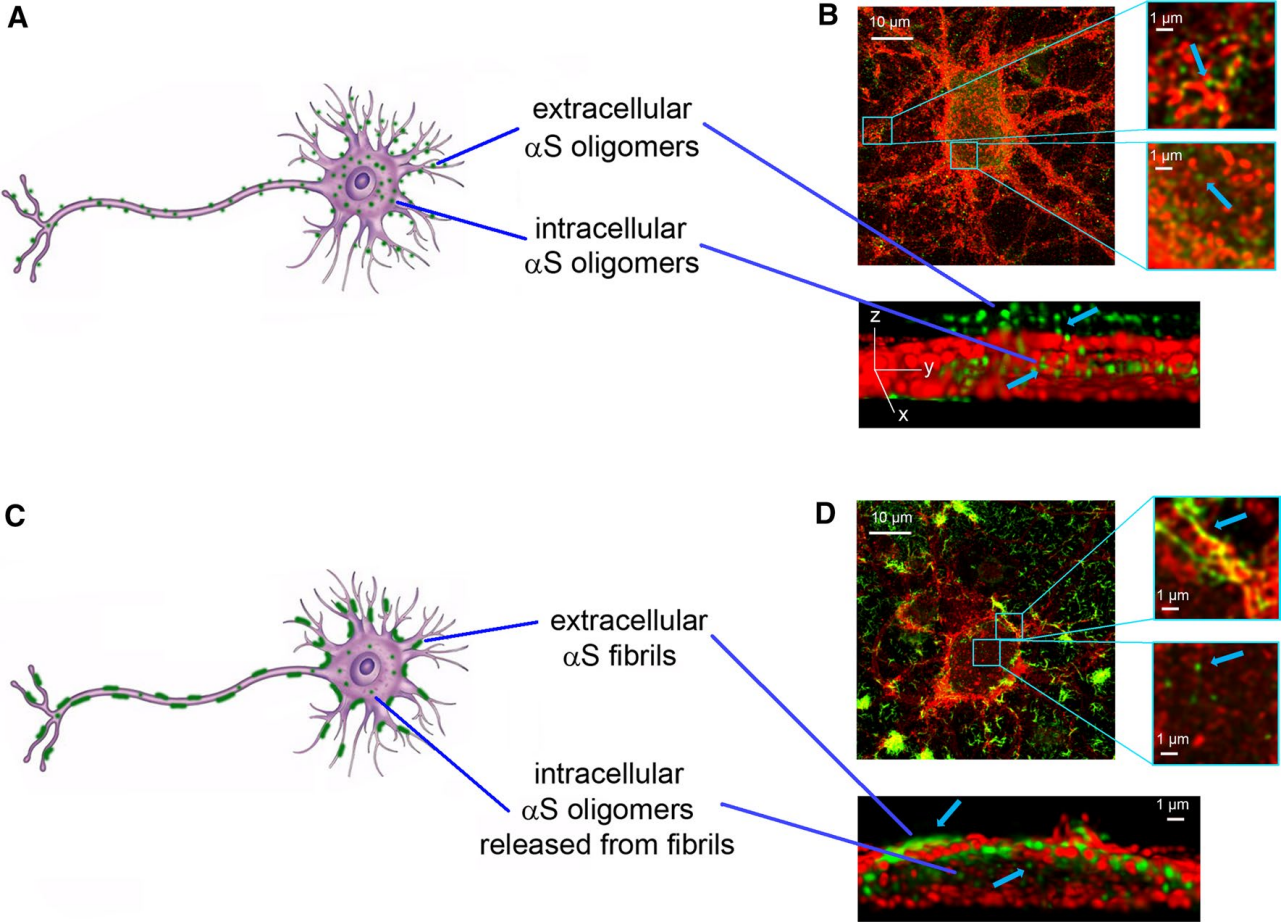
The release of αSyn oligomers from mature fibrils. Schematic representation of neurons exposed to αSyn oligomers (**A**) and fibrils (**C**). Representative STED images of primary rat cortical neurons treated with preformed oligomers (**B**) and fibrils (**D**) with higher magnifications in the boxed areas on the right. Red and green fluorescence indicates the cell membranes and the αSyn species, respectively. A 3D reconstruction of a primary neuron on the *zy* plane shows the extracellular (top) and intracellular (middle) αS species. STED images reprinted from [11], licensed under Creative Commons Attribution 4.0 International Public License (CC BY 4.0,https://creativecommons.org/licenses/by/4.0/)

We have recently demonstrated that the toxic effects of the fibrils are directly correlated with the amount of αSyn that penetrate into the neuronal cells after interacting with the cellular membranes and cause calcium uptake, ROS formation, membrane permeabilization, caspase-3 activation and mitochondrial dysfunction [11]. In particular, we provide further evidence for the release of toxic oligomeric species from fibrils, upon extracellular administration of αSyn fibrillar species to neuronal cultures, using specific probes able to distinguish them from their fibrillar precursors [11, 157], (Fig. 2).

According to our results, the dissociation of αSyn fibrils into soluble αSyn species, likely to include a low proportion of monomers in addition to oligomers, have also been observed in recent studies to occur under conditions close to physiological [12, 158]. These conclusions are in line with previous reports obtained with the amyloid beta (Aβ) peptide associated with Alzheimer’s disease that have revealed the lipid-mediated depolymerization of non-toxic fibrils of Aβ into toxic A11-positive oligomers, which were also shown to resemble the oligomers formed de novo during fibril assembly [159]. They are also in agreement with the general proposition that any fibrillar species that accumulate in tissue can represent a source of soluble toxic oligomers [12, 160], and with the halos of soluble oligomers observed to surround amyloid plaques of Aβ in mouse brains [161].

This evidence suggests that αSyn fibrils, in addition to their ability to transfer from neuron-to-neuron contributing to the progressive diffusion and spreading of LB pathology in different brain areas [15, 17, 57, 124, 143, 162–164], can release prefibrillar oligomeric species that cause an immediate dysfunction of the neurons in the vicinity of these species [11, 157] (Fig. 2). Such released oligomeric species could also contribute to pathogenesis via neuron-to-neuron spreading by their direct cell-to-cell transfer or by generating new fibrils, following their neuronal uptake.

## Fibril spreading

### αSyn, a prion-like protein

Prions were firstly defined by Stanley Prusiner as “proteinaceous infectious particles” able to transmit cell-to-cell, thus causing the propagation of specific diseases [165]. This term arises from the misfolding of endogenous native cellular prion protein (PrP^C^) into a pathogenic conformation referred to as scrapie (PrP^Sc^), able to recruit and corrupt PrP^C^, thus inducing the formation of misfolded conformers with self-propagating capacities [166]. Similarly, several proteins involved in the most widespread neurodegenerative diseases, such as αSyn [15], tau [167], amyloid-β [168] and Huntingtin [169], were extensively reported to adopt a similar spreading mechanism.

Staging studies of PD support the prion-like nature of αSyn, as its aggregated forms appear in the brain following a well-defined spatially and temporally stereotyped fashion [170–173]. The so-called Braak hypothesis posits that αSyn from LBs and LNs spreads between synaptically connected brain areas [172–174]. This transmission would be responsible for the propagation of the pathology and strongly correlates with the progression and the severity of the disease-associated symptoms. According to this theory, Lewy pathology is initiated by an undefined pathogen (virus or bacterium) that enters in the nasal cavity, and subsequently reaches the gut [173], the pathogen spreads via the olfactory tract or the vagus nerve, joining the central nervous system (CNS, and in particular the medulla oblongata and the olfactory bulb), thus causing deficits in the sense of smell, that are early markers of the preclinical phases of PD (Braak stages 1 and 2); the propagation carries on into the brainstem, thus inducing sleep and motor disturbances (Braak stages 3 and 4), then into the limbic system, finally reaching the neocortical regions, determining cognitive impairment and emotional disturbances in Braak stages 5 and 6 [175]. Braak staging of PD is supported in vitro, in vivo, and by the vast majority of clinical cases, in particular those with early onset and longest disease duration [176]. Many studies conducted both in humans and model systems clearly indicate that αSyn aggregates can be released from neurons [177], and subsequently taken up by the nearby, thus inducing Lewy pathology. Furthermore, αSyn is largely present, in different aggregated forms, in biological fluids such as CSF, plasma, and saliva [105, 177, 178]. In addition, clinical evidence from post-mortem analysis of PD patients who received donor nigral grafts showed that more than 10 years after, Lewy pathology was also present in healthy donor neurons, indicating αSyn spreading from unhealthy neurons of the host brains [147, 148].

When αSyn preformed fibrils (PFFs) were injected into the brain of transgenic mice overexpressing human A53T αSyn, αSyn aggregation and LBs formation were massively exacerbated by the uptake of PFFs; these aggregates were also injected in mice brains overexpressing the wild-type protein, and LB-like inclusions were found to spread through the mouse brain following a precise spatiotemporal pattern [15]. Many other investigations have been performed in animal models such as mice [16, 143, 179] and rats [144]. It has been shown that small fibrillar aggregates are particularly effective in seeding and spreading when injected into non-transgenic mouse brains, while long fibrils are largely inefficient [57], suggesting that oligomers with high seeding capabilities might also be implicated in αSyn pathology propagation. Some studies have reported that different types of αSyn oligomers are capable to seed αSyn aggregation in cells and propagate αSyn pathology [68, 180]. However, other studies trying to analyse the role of oligomeric species in this process have used in vitro generated/stabilised αSyn oligomers, which typically show impaired elongation/seeding properties as a consequence of the protocols used to trap them [21, 54, 57]. In all cases, the injection of αSyn aggregates induces the formation of LB-like inclusions, thus leading to the spreading of αSyn pathology and, as a consequence, to the death of vulnerable neuronal populations, thus recapitulating the main features of PD. Collectively, these evidences suggest that αSyn aggregates are able to self-propagate and spread in a prion-like manner, acting as seeds, and inducing the misfolding of soluble native αSyn, thus initiating pathology in recipient neurons. Importantly, the synaptic connection between different brain regions is necessary but not sufficient for causing neuronal cell death, that is strongly associated with the intrinsic vulnerability of specific neuronal populations [181]. Evidences obtained in the aforementioned animal models also indicated that the distribution of pathological lesions in the brains of injected rodents are strictly associated with the biophysical, structural and biochemical characteristics of the assemblies, thus corroborating the hypothesis of the existence of distinct αSyn conformers in vivo. This is consistent with the proposition of the prion-like behaviour of αSyn, as prions exist in distinct stable conformations, commonly referred to as strains, possessing distinct but relevant pathological, clinical, and diagnostic implications. [106, 121, 123, 124, 182]. Recently, filament preparations from MSA brains were used for the in vitro seeded assembly of recombinant human α-synuclein [183]. The researchers found that the structures of the seeded assemblies differ from those of the seeds, suggesting that additional, as yet unknown, factors play a role in the propagation of the seeds [183]. Thus, identification of these factors will be essential for understanding the prion-like spreading of α-synuclein proteinopathies.

### The release of αSyn fibrils

In the last decades, major efforts have been performed to clarify the spreading mechanism of αSyn fibrils between interconnected vulnerable brain regions and Lewy pathology propagation, and a range of different cellular and animal models have been used to explain its transmission.

The ability of neurons to excrete αSyn monomers, oligomers, and fibrils through non-conventional calcium-dependent exocytosis from vesicles or exosomes has been widely described [79, 184]. In particular, Lee et al. [185] reported a misfolding-associated protein secretion pathway which uses the endoplasmic reticulum (ER)-associated deubiquitylase USP19 to export aberrantly aggregated proteins, including αSyn. Concomitantly, Fontaine and coworkers showed that the co-chaperone DnaJC5, in complex with the chaperone Hsc70, are critically involved in the release of neurodegenerative disease proteins αSyn, tau and TDP-43 [186]. However, it is not clear if these two pathways are connected and if they are involved in the release of oligomeric or fibrillar forms of αSyn. Importantly, Hsc70 was previously reported to bind αSyn fibrils with much major affinity as compared to the soluble protein in vivo, and that the neurotoxicity of fibrillar conformers coated with Hsc70 was significantly minor with respect to that of uncoated ones, suggesting a possible role of this chaperone in reducing the spreading of αSyn fibrils throughout the CNS [151].

The release of αSyn fibrils from neurons has been investigated by taking advantage of microfluidic cell culture chambers, sophisticated tools allowing the separation of neuronal processes from somata via a series of interconnected microgrooves, in fluidically isolated channels [187]. Volpicelli-Daley and coworkers showed that αSyn PFFs were internalized when added to the neuritic chamber, or either to somata, of primary hippocampal neurons, resulting in insoluble phospho-αSyn-positive aggregates in different cellular compartments, resembling those found in the brain of people affected by synucleinopathies [17]. Fluorescently labelled αSyn PFFs were also reported to be internalized by primary neurons, transported in axons and rapidly observed in the cell bodies of immature second-order neurons following anterograde axonal transport [188], the secretion of αSyn fibrils occurs in the absence of axonal lysis, suggesting that their propagation from neuron to neuron occurs in healthy cells [162]. This observation is consistent with that obtained in vivo in a rat model overexpressing αSyn in the medulla oblongata, in which αSyn release from degenerated neurons did not result to cause a significant spreading to more rostral brain areas; by contrast, αSyn propagation was significantly more pronounced in intact healthy neuronal cells [189]. Using a three-chamber microfluidic device, Mao and coworkers reported that PFF added to the first chamber can transmit and induce the aggregation of endogenous αSyn in synaptically connected neurons in the other two chambers [190]. Consistently, the addition of specific αSyn monoclonal antibodies to the culture medium blocked the entry of PFFs in primary neurons, and the subsequent spreading of αSyn pathology to the nearby cells. Accordingly, the administration of such antibodies to a wild-type mouse previously inoculated with PFFs was able to reduce αSyn spreading, neuronal loss, and the associated motor dysfunction triggered by αSyn pathology [191].

Tunneling nanotubes (TNTs), F-actin-containing membranous channels connecting two or more remote cells [192], have been reported to play a major role in the transmission of αSyn fibrils between neurons. Abounit and coworkers revealed for the first time that αSyn fibrils directed to lysosomes for being degraded, were able to transfer between neuronal cells inside lysosomal vesicles via TNTs, and to induce the aggregation of endogenous αSyn [193]. Accordingly, lysosomal impairment was described to increase αSyn transfer between cells [194]. Recently, the mechanism by which αSyn fibrils spread through lysosomes was described in more detail: they were reported to alter lysosomal morphology and functionality, and to induce the peripheral redistribution of lysosomes, thus increasing their transfer to neighboring cells [195]. The transmission of αSyn fibrils through TNTs was also observed by Dieriks and collaborators in SH-SY5Y cells and in primary human brain pericytes derived from postmortem PD brains, pointing out the central role of non-neuronal cells in PD progression [196].

Importantly, other amyloidogenic proteins, including the prion protein [193, 197, 198], tau [197, 199, 200], and huntingtin [201] were previously described to transfer between cells through TNTs, suggesting that these cellular interconnections are primarily involved in the pathogenic spreading of misfolded protein aggregates between cells, and that they could be considered as suitable targets for therapeutic intervention in α-synucleinopathies and other pathologies in which protein aggregation and prion-like spreading of misfolded aggregates is linked to neurodegeneration.

### The uptake of αSyn fibrils

The uptake of αSyn fibrils from the extracellular space has been extensively investigated in recent years, as it is considered a crucial event resulting in neurotoxicity and seeding of the endogenous protein. Many research groups are focused on the uptake of αSyn fibrillar conformers by neurons, and constantly report significantly different kinetics, extent and mechanisms of uptake, given the use of diverse cellular models, culture conditions and aggregation protocols for fibril production, giving rise to distinct fibrillary strains with heterogeneous structural, biophysical and biological properties. Despite these differences, there is strong evidence of fibril internalization both in cultured cells [14, 17, 162, 193, 202, 203] and in vivo, in mouse models following the stereotactical injection of αSyn fibrils into the olfactory bulb: fibrils are uptaken by neurons and glia neighbouring the injection site [16].

αSyn fibrils bind to the extracellular leaflet of plasma membranes, and then gain entry into the cytosol of neuronal cells through different mechanisms, such as endocytosis, macropinocytosis, or by interacting with specific membrane receptors [204]. Among them, endocytosis has been widely investigated. Is has been demonstrated that the inhibition of dynamin-dependent endocytosis, either by low temperature, or through the expression of a dominant-negative mutant of dynamin-1, markedly decreases fibril internalization in different cell lines, including primary neurons [76, 163, 184, 193]. Very similar results were obtained when neuronal cells were treated with the dynamin inhibitor Dynasore [205]. The lymphocyte activation gene-3 (Lag-3), a member of the immunoglobulin superfamily of receptors, was reported to bind αSyn PFFs and to mediate their clathrin-mediated endocytosis. Accordingly, Lag-3 knockout reduced αSyn spreading in primary neurons and in mice CNS, thus preventing the subsequent aggregation of endogenous αSyn [190]. Importantly, the reduction was non total, and in the same study Mao and coworkers revealed that the Ab precursor-like protein 1 (APLP1) also act as a receptor leading to the entry of αSyn fibrils [190]. In very recent work, Zhang and coworkers investigated in more detail the molecular mechanism underlying the selective binding of fibrillar αSyn to neuronal cells through Lag-3 and APLP1 receptors, revealing that the acidic C-terminus of αSyn, particularly exposed on the surface of fibrillar conformers, is the key domain binding to a positively charged surface on the analysed receptors [206]. Importantly, S129 phosphorylation of αSyn, a typical modification of pathological αSyn abundant in Lewy bodies and neurites, determines a significant increase in the binding of αSyn fibrils to the receptors, thus promoting their spreading, finally culminating in neurodegeneration [206].

Over the last decade, heparan sulfate proteoglycans (HSPGs) emerged as a receptor and specific mediator for the uptake of αSyn, tau and Aβ fibrils by macropinocytosis [207–209]. Such pathway occurs in neurons and oligodendrocytes, but not in immune cells of the brain like astrocytes and microglial cells, that probably employ different internalization mechanisms [210]. Accordingly, heparin was able to inhibit the binding on the cell surface and the internalization of αSyn fibrils [207], but this anticoagulant is not suitable for the chronic treatment of PD. Other researchers used a proteomic approach for the identification of other possible membrane interactors of αSyn fibrils, and they found that the α3-subunit of Na^+^/K^+^-ATPase (α3-NKA) interacted with fibrillar, but not monomeric or oligomeric forms of αSyn [211]. Accordingly, mutations in the gene encoding α3-NKA (ATP1A3), has been associated with rapid-onset dystonia-parkinsonism [212]. The prion-like cell-to-cell transmission of fibrillar αSyn has been recently reported to be also mediated by the Fc gamma receptor IIb (FcγRIIB), an inhibitory receptor physiologically present in the surface of neuronal cells, where it binds immunoglobulins G with low affinity [213, 214]. The same receptor was also described for its ability to induce the accumulation of amyloid-β oligomers [215], thus representing a common ligand for the neuropathic uptake of misfolded conformers in neurodegenerative diseases.

Mounting evidence suggests that PrP^C^ is partially responsible for the uptake of αSyn fibrils [216]. Indeed, Aulić and collaborators reported that PrP^C^ promotes the internalization of αSyn fibrils both in cultured cells and in vivo, in wild type (*Prnp*^+^*/*^+^) mice as compared to PrP knock-out (*Prnp*^−^/^−^) animals through the direct binding by its N-terminal domain [216]. PrP^C^ was also reported to facilitate the entry of tau [217] and TDP-43 [218] fibrils in neuronal cells, as well as to interact with αSyn [89] and amyloid-β oligomers [219] and mediate their toxic downstream effects. Thus, PrP^C^ is widely considered as a common cellular conformation-specific sensor for disease-associated proteins involved in neurodegeneration. Taken together, these evidences suggest that the reduction of cell-to-cell transmission of fibrillar αSyn could be a considerable therapeutic target to limit the disease to certain brain regions where inclusions are already present, thus avoiding their spreading. However, the plethora of possible mechanisms leading to the pathological transmission of harmful αSyn conformers, makes this challenge very complex.

Once internalized, αSyn fibrils are surrounded by endocytic membranes and directed to late endosomal compartments and lysosomes, without having a direct access to the wild-type, cytosolic, soluble αSyn [76, 193, 202]. Consistently, Sacino and coworkers [205] observed that when αSyn PPFs were added to the culture medium of neuronal-glial cells, they were progressively degraded, and that the inhibition of the lysosomal activity caused the accumulation of PPFs in vesicles. Interestingly, Loria and coworkers also reported lysosomal degradation of αSyn fibrils in astrocytes and, less efficiently, in neurons, suggesting a prominent role of astrocytes in clearing αSyn pathological deposits [220].

**Fig. 3:**
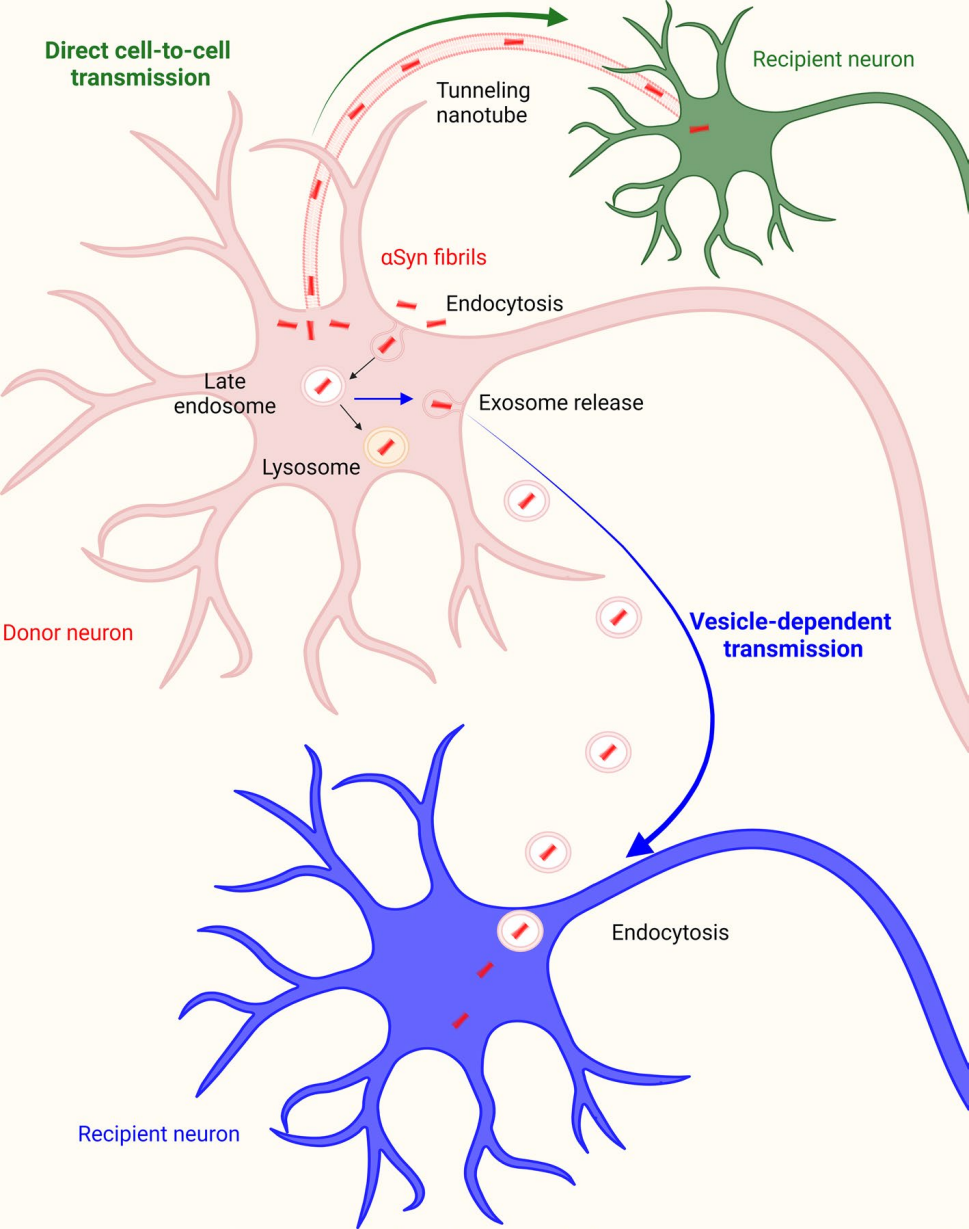
αSyn fibrils spreading. αSyn fibrils are internalized by neuronal cells through endocytosis. Then, they reach the lysosomal compartment by travelling through early and late endosomes. αSyn fibrils escape the endosomal compartment through an undefined mechanism, and are released via exocytosis or tunneling nanotubes (TNT), thus reaching neighboring neurons and spreading the pathology. Created with BioRender.com

The precise escapement mechanism from the lumen of endocytic vesicles and the subsequent colocalization of αSyn fibrils with the endogenous αSyn are still a matter of debate, but this process was proposed to occur thanks to their ability to disrupt the integrity of endocytic membranes [221]. This evidence suggests that exogenous αSyn assemblies could act as nucleating seeds, able to recruit intracellular αSyn, giving rise to the formation of large insoluble LB-like inclusions [14, 17], imprinting their structural characteristics onto endogenous protein, and so causing its misfolding [123, 124]. An overview of the major molecular mechanisms responsible for the transmission of αSyn fibrils is given in Fig. 3. The so formed LB-like aggregates cause the global decrease of synaptic proteins, progressive impairments in neuronal excitability and connectivity, finally culminating in cell death [17]. These structures were found to be insoluble in detergent, hyperphosphorylated, ubiquitinated, and characterized by a filamentous ultrastructure revealed by electron microscopy [222].

## Concluding remarks

**Fig. 4:**
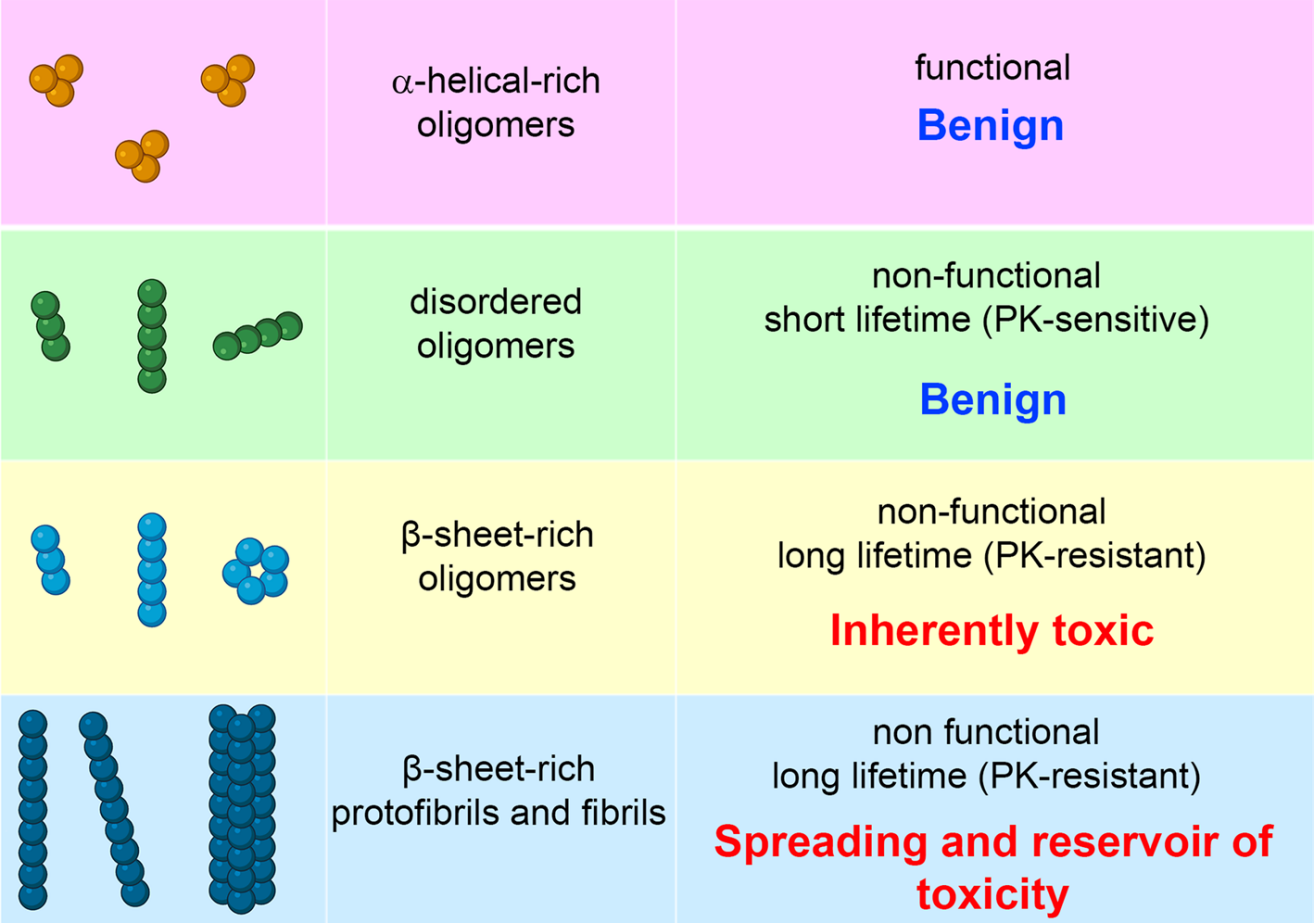
Scheme of the main structural and biological features of aSyn species. Created with BioRender.com

Protein misfolding and aggregation process in the cell can generate an ensemble of oligomers with differing β-sheet arrangements, rates of elongation and pathological roles (Fig. 4), depending on the nature of the biological processes occurring during the self-assembly pathway. In addition, the elongation prone, fibril-like oligomers with parallel β-sheet arrangement could act as pathogenic species for the spreading and transmission of the disease, whereas long-lived and protease-resistant oligomers with an antiparallel β-sheet arrangement could accumulate within cells and act as potent toxins. Recent NMR and CD data show that the β-sheet core of the αS fibrils is unable to establish persistent interactions with the lipid bilayers, but they can release oligomeric species responsible for an immediate dysfunction of the recipient neurons. Reversibly, such oligomeric species could also contribute to pathogenesis via neuron-to-neuron spreading by their direct cell-to-cell transfer or by generating new fibrils, following their neuronal uptake. In conclusion, different types of aggregates populated during the process of αS self-assembly appear to be involved with different molecular mechanisms in synucleinopathies: soluble oligomers are directly implicated in the induction of neurotoxicity and fibrils in the propagation of pathology.

## Data Availability

Enquiries about data availability should be directed to the authors. Data sharing not applicable to this article as no datasets were generated or analysed during the current study
